# Exposure to different lengths of sick leave and subsequent work absence among young adults

**DOI:** 10.1186/s12889-015-2679-0

**Published:** 2016-01-20

**Authors:** Magnus Helgesson, Bo Johansson, Lisa Wernroth, Eva Vingård

**Affiliations:** 1 Department of Medical Sciences, Occupational and Environmental Medicine, Uppsala University, Ulleråkersvägen 40, 751 85 Uppsala, Sweden; 2 Occupational and Environmental Medicine, Uppsala University Hospital, Uppsala, Sweden; 3 Uppsala Clinical Research Centre, Uppsala University, Uppsala, Sweden

**Keywords:** Sick leave, Unemployment, Disability pension, Immigrants, Social insurance, Work absence

## Abstract

**Background:**

Sweden has a public and easily accessible sickness insurance. Research shows, however, downsides to taking sick leave. Both short and longer periods of sick leave have been seen to increase the risk for subsequent work absence. The aim of this study was to investigate whether there was an association between sick leave claimed in 1993 and work absence in the subsequent 15 years, i.e. up to 2008. A further aim was to explore differences in this relation with regard to gender, origin and educational level at baseline.

**Methods:**

Our cohort consisted of all immigrants aged 21–25 years in Sweden in 1993 and a control group of native Swedes in the same age group.

**Results:**

Subsequent work absence increased from 313 days among persons with no days of claimed sick leave in 1993 to 567 days among persons with 1–7 days of claimed sick leave in 1993. Thereafter there was a lower, but steady increase in days of future work absence, to 611 days among persons with 8–14 days of sick leave claimed in 1993. There was an interaction between sick leave and gender, education and origin respectively regarding later work absence.

**Conclusion:**

Periods of sick leave claimed were associated with subsequent work absence. Immigrants, women and persons with low education had the most risk of future work absence after a period of sick leave.

## Background

Sick leave has been reported to increase the risk for later work absence and even permanent exclusion from the labour market [1]. Persons with longer spells of sick leave, but also persons on repeated short spells of sick leave, have an increased risk for additional work absence [2, 3]. Persons on sick leave further have an increased risk of unemployment [4]. Most individuals on sick leave have non-fatal diseases, most often musculoskeletal disorders or common mental disorders [5]. Still, persons on sick leave for musculoskeletal problems not only have an elevated risk of later work absence due to sickness absence, disability pension and unemployment, but are also at increased risk of dying prematurely [6]. This shows an intricate relationship between sick leave and later attachment to the labour market as well as later health status, implying that something beyond the illness itself may cause the adverse outcomes on work and health [3].

In assessing the consequences of sick leave it is important also to know whether there are differences between groups. In Sweden since the 1980s, there have been an increasing proportion of women on sick leave. Today women account for around two-thirds of all days of sick leave [5]. Furthermore, high educational level has been seen to mediate the risk for sick leave and one reason may be that persons with low education have physically more demanding jobs [7]. Compared with many other countries, Sweden has a relatively large and growing immigrant population. Both unemployment and disability pension is more common among immigrants than in the native population, both in Sweden and in Norway [8–10]. Therefore it is of importance to scrutinise if there are any differences in subsequent work absence after a period of sick leave in immigrants compared to that of native Swedes.

Previous research has most often used either health related measures or labour market measures when assessing the consequences of sick leave [3, 4, 11]. There is therefore a need for studies that address total work absence, and not analyses them separately. Taking only one of the measures of subsequent sick leave, unemployment and disability pension into consideration when measuring the consequences of sick leave may underestimate the social consequences of sick leave and miss uneven access between groups to various parts of social security [12]. Previous studies on this subject have focused on either short periods of sick leave [3], or longer periods of sick leave [13]. There is also a need for studies that assess a broader spectrum of sick leave in order to gain a better understanding of the relation between sick leave and subsequent absence from work.

## Aims

The main aim of the study was to assess the impact on later work absence regarding the length of sick leave claimed in 1993. A further aim was to explore whether there were differences in the pattern of later work absence between subgroups categorized by gender, education (elementary, upper secondary, university) and origin (immigrant or native Swede).

## Methods

### Exposure and outcome variables

The exposure variable was sick leave, which in our study meant that a sickness benefit claim from the Social Insurance Agency had been made. In Sweden, the employer has covered at least 14 days of all sick leave spell during the study period (until 31 December 1996: 14 days; 1 January 1997 to 31 March 1998: 28 days; 1 April 1998 to 30 June 2003: 14 days; 1 July 2003 to 31 December 2004: 21 days; After 1 January 2005: 14 days). This period is not visible in any public register for persons with an employer. After 7 days of sick leave, a doctor has to certify that the person’s work ability is reduced. Disability pension, sick leave and unemployment benefit are regulatory mutually exclusive. It is, however, possible to get sickness benefit also when unemployed, but you will then be unregistered as jobseeker.

The outcome variable in this study was total days of work absence during a follow-up period of 15 years. Total work absence was summed up based on (1) whole days of unemployment registered at the National Employment Agency; (2) net days of sick leave registered at the Social Insurance Agency; and (3) days on disability pension registered at the Social Insurance Agency.

### Study population

This study was a prospective cohort study based on registers. The study group comprised all immigrants aged 21–25 years who were living in Sweden in 1993 and had immigrated to the country before 1990 (*n =* 14,423). A random sample of native Swedes of the same age group were also included (*n =* 121,084) (Table 1). In this study the term “immigrant” refers to a person born outside Sweden with two non-Swedish-born parents. “Native Swede” refers to a person born in Sweden with two Swedish-born parents. Further, all immigrants are referred to as one group. The cohort was followed from 1994 to 2008. We wanted a relatively healthy cohort, so we excluded the following persons from the analyses: individuals who received disability pension from 1990 to 1993, and individuals who were hospitalized between 1990 and 1993 with a pulmonary, cardiovascular, musculoskeletal or psychiatric diagnosis. We also excluded individuals who were on unemployment in 1993 in order to reduce the number of persons with register data on sick leave from day 1 already. Persons that died during the follow-up were excluded at the time of death. Individuals who emigrated from Sweden during the follow-up were removed from the study because we had insufficient data concerning their whereabouts.

**Table 1:** Study population

		N (%)
	All	135,507
Sex	Men	65,335 (48,2 %)
	Women	70,172 (51,8 %)
Origin	Immigrant	14,423 (10,6 %)
	Native Swedish	121,084 (89,4 %)
Education	Elementary	14,783 (11,0 %)
	Upper secondary	90,212 (66,9 %)
	University	29,902 (22,1 %)
Sick leave 1993	0 days	120,226 (88,7 %)
	1–7 days	4,861 (3,6 %)
	8–14 days	2,309 (1,7 %)
	15–28 days	2,519 (1,9 %)
	29–59 days	2,449 (1,8 %)
	60–89 days	1,038 (0,8 %)
	90–119 days	595 (0,4 %)
	120–149 days	309 (0,2 %)
	150–179 days	228 (0,2 %)
	180–239 days	293 (0,2 %)
	240–299 days	180 (0,1 %)
	300–364 days	253 (0,2 %)
	365 days	247 (0,2 %)

### Statistics

In order to describe the association between sick leave in 1993 and work absence during 1994–2008, sick leave was categorized into 13 classes. Mean and median work absence, with 95 % confidence intervals (CIs) based on the normal distribution, was calculated within each class. The association between sick leave during 1993 and work absence during 1994–2008 was analysed using the zero-inflated negative binomial (zinb) model. To investigate whether gender, education and origin have an impact on the association between sick leave and work absence, models including pairwise interactions with sick leave were estimated. Because of the low number of individuals on sick leave for ≥120 days among individuals with a university education and immigrants, the interaction analyses including immigrants and university-educated persons were limited to individuals with sick leave up to 90–119 days.

Sick leave in 1993 was included in the models as a class variable (0, 1–7, 8–14, 15–28, 29–59, 60–89, 90–119, 120–149, 150–179, 180–239, 240–299, 300–364 and 365 days) and all models were adjusted for age and residential area. We used gender, age, country of origin, and education as inflation variables in the logit model for predicting excess zeros (see Appendix 1). We applied the Bonferroni adjustment for multiple comparisons. For the seven pairwise tests comparing immigrants with native Swedes, the differences were statistically significant if *p <* 0.007. For education and gender, the differences were considered statistically significant if the p-value was <0.004. The statistical analyses were performed using SAS version 9.3 (SAS Institute Inc., Cary, NC, USA).

### Ethics approval

The study was approved by The Regional Ethical Review Board in Uppsala, Sweden; file number 2009/134.

## Results

### Total

**Fig. 1:**
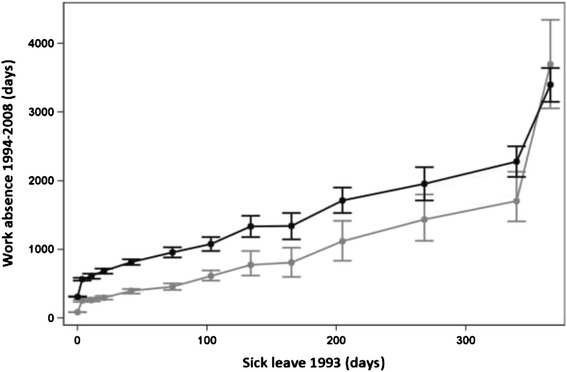
Relation between accumulated days of sick leave in 1993 and work absence 1994–2008. Relation between mean (black) and median (grey) accumulated days of sick leave in 1993 and number of days of work absence during 1994–2008, with 95 % confidence intervals (CIs) shown. The points in the graph refer to 0, 1–7, 8–14, 15–28, 29–59, 60–89, 90–119, 120–149, 150–179, 180–239, 240–299, 300–364 and 365 days of sick leave taken in 1993

There was a steep increase in future work absence, from 313 days on average among persons with no days of sick leave to 567 days on average among persons with 1–7 days of sick leave claimed (Fig. 1). Thereafter, the increase was more gradual up to 611 days of work absence among persons with 8–14 days of sick leave claimed. There was another steep rise after 300 days of sick leave claimed, from 2,283 days of work absence among persons with 300–364 days of sick leave to 3,398 days’ future work absence among persons with 365 days of sick leave claimed. In the dataset, most individuals had low values on later work absence, and hence the median value was lower than the mean value.

### Gender

**Fig. 2:**
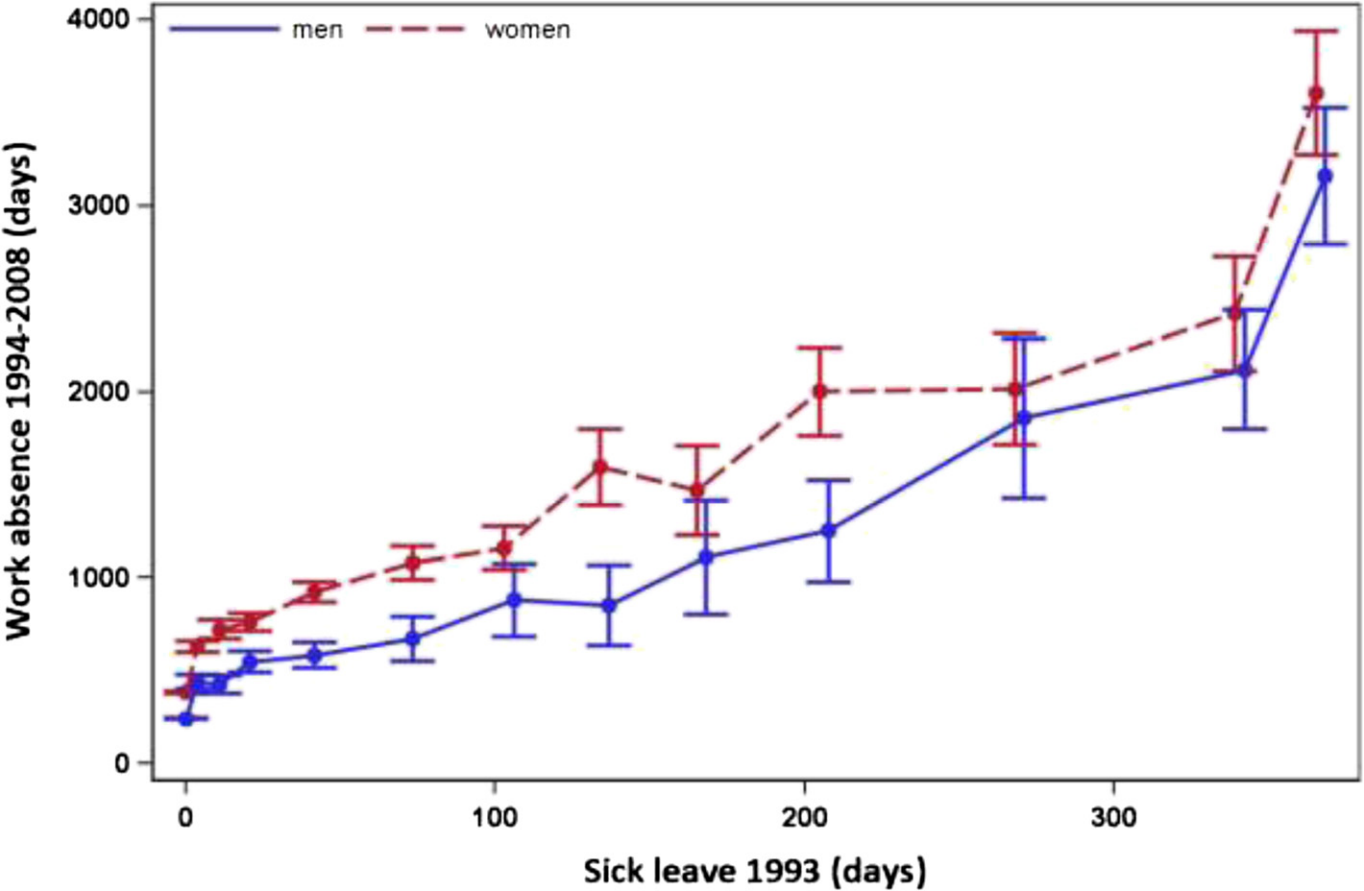
Accumulated days of sick leave in 1993 and later work absence 1994–2008, by gender. Relation between accumulated days of sick leave in 1993 and mean number of days of work absence during 1994–2008, by gender, with 95 % confidence intervals (CIs) given. The points in the graph refer to 0, 1–7, 8–14, 15–28, 29–59, 60–89, 90–119, 120–149, 150–179, 180–239, 240–299, 300–364 and 365 days of sick leave taken in 1993

Sick leave was associated with significantly more days of later work absence among women compared with men for every given level of sick leave claimed up to 120–149 days, with the exception of individuals with 90–119 days of sick leave claimed (p value for interaction = 0.011) (Fig. 2). Women with no sick leave in 1993 on average had 139 days more days of subsequent work absence compared with men with no sick leave in the same year. The difference between men and women increased to 403 days for persons with 60–89 days of sick leave claimed and 745 days among persons with 120–149 days of sick leave claimed.

### Education

**Fig. 3:**
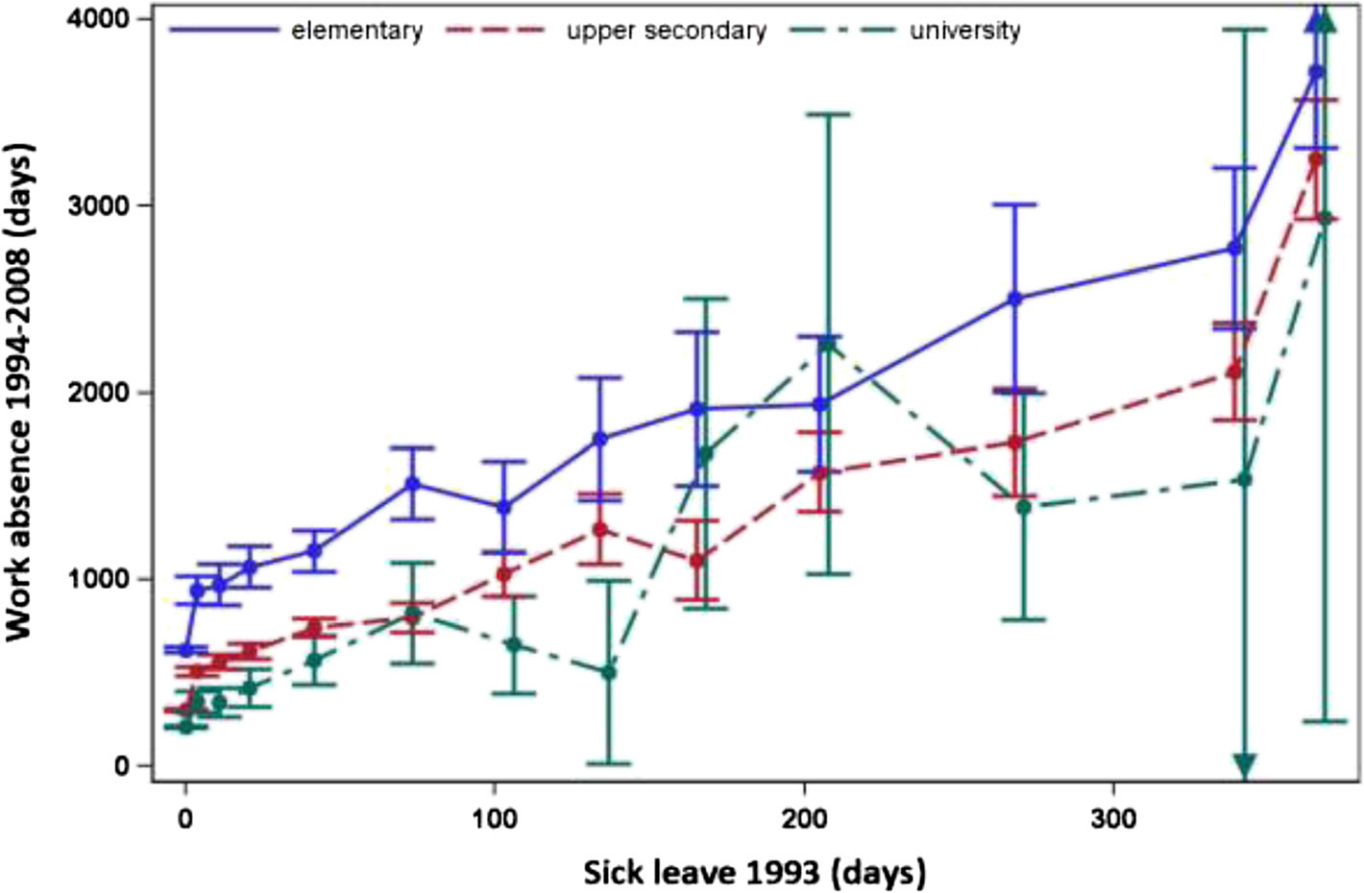
Accumulated days of sick leave in 1993 and later work absence 1994–2008, by education Relation between accumulated days of sick leave in 1993 and mean number of days of work absence during 1994–2008, by educational level, with 95 % confidence intervals (CIs) shown. The points in the graph refer to 0, 1–7, 8–14, 15–28, 29–59, 60–89, 90–119, 120–149, 150–179, 180–239, 240–299, 300–364 and 365 days of sick leave taken in 1993

Individuals with only an elementary school education had more days of work absence for every given level of sick leave claimed up to 90–119 days compared with persons with a university education and up to 60–89 days of sick leave claimed compared to persons with upper secondary education (p value for interaction <0.001) (Fig. 3). The difference in days of work absence for individuals with elementary school compared with persons with upper secondary school education ranged from 326 days for persons with no sick leave to a maximum of 716 days among individuals with 60–89 days of sick leave claimed in 1993. Compared with persons with a university education, persons with elementary school education had from 397 to 694 days more work absence in the follow-up period depending on length of sick leave claimed in 1993. Among those who had completed upper secondary school, individuals with no sick leave in 1993 on average had 91 days more days of work absence compared with individuals with a university education. The difference was greatest, 218 days, among persons who claimed 8–14 days of sick leave in 1993.

### Origin

**Fig. 4:**
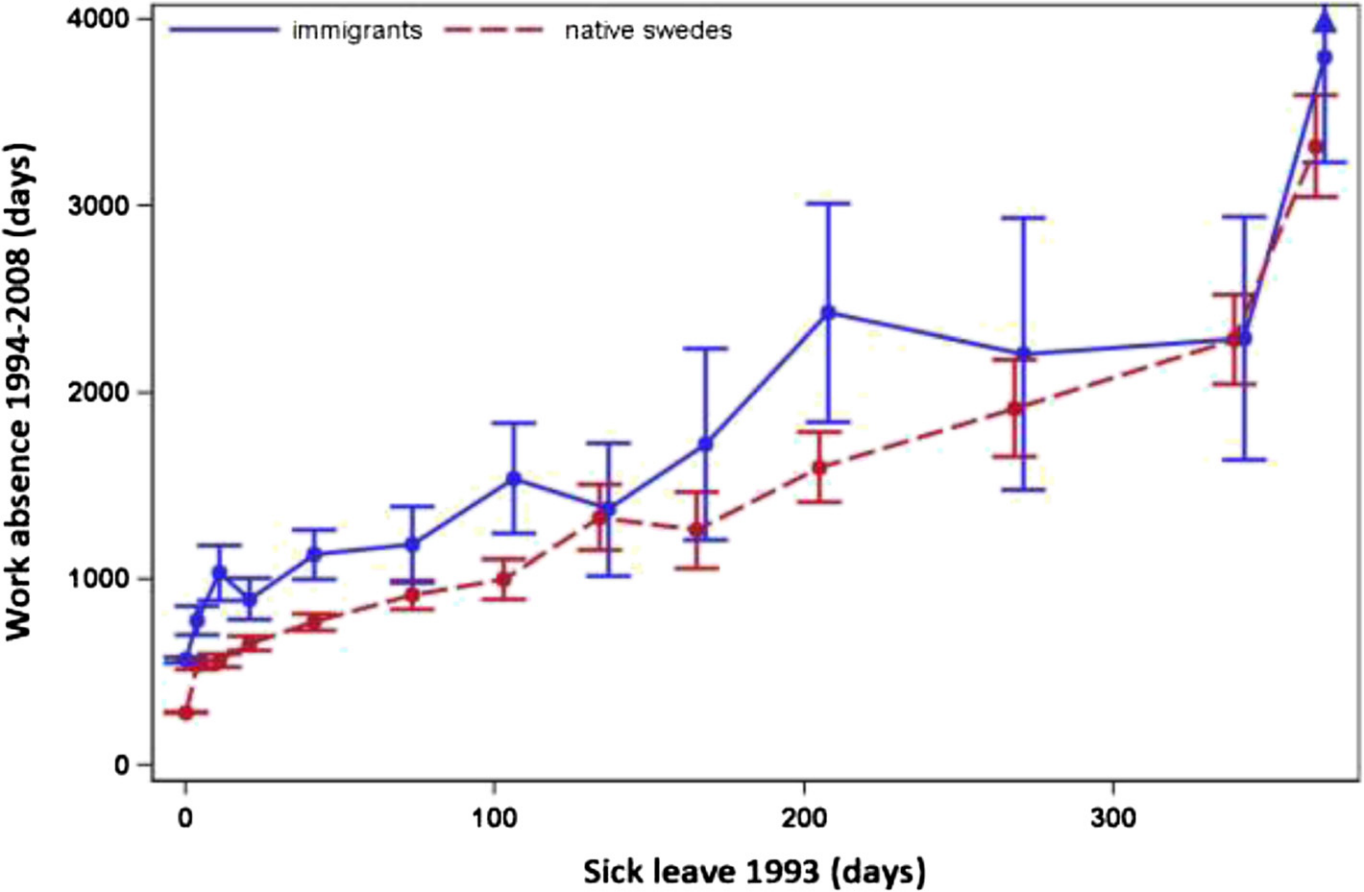
Accumulated days of sick leave in 1993 and later work absence 1994–2008, by country of origin. Relation between accumulated days of sick leave in 1993 and mean number of days of work absence during 1994–2008, by individuals’ country of origin, with 95 % confidence intervals (CIs) given. The points in the graph refer to 0, 1–7, 8–14, 15–28, 29–59, 60–89, 90–119, 120–149, 150–179, 180–239, 240–299, 300–364 and 365 days of sick leave taken in 1993

Sick leave was associated with significantly more days of later work absence among immigrants compared with native Swedes for every given level of claimed sick leave up to 29–59 days (p value for interaction <0.001) (Fig. 4). The difference seemed to fluctuate, with no obvious increase in work absence for every increase in sick leave claimed in 1993. Immigrants had between 220 and 469 more days of work absence in the follow-up period compared with native Swedes.

## Discussion

Exposure to sick leave in 1993 was associated with subsequent work absence. There were differences regarding gender, educational level at baseline and immigrant status. There were also differences regarding length of sick leave taken in 1993. A dose–response relationship was seen, but with different rates of increase with respect to length of sick leave in 1993. There was a steep increase in later work absence for persons who took 1–7 days of sick leave claimed, compared with persons with no sick leave, which then levelled off with further sick leave taken.

The reasons for an increase in the risk for future work absence for persons with spells of sick leave seem to be multi-faceted, with many factors involved in the process, from individual abilities to societal structures. The vast majority of sicknesses will heal naturally without any intervention. Short spells of sick leave should therefore, from a health perspective, not be decisive for future work absence. It is, however, important to prevent short-term or repeated short spells of sick leave from leading to long spells of sick leave [2, 3]. In our study cohort, there was a steady increase in future days of work absence with an increase of sick leave taken in 1993. The concept of “sick role” was introduced by Parsons in the mid-20th century and focuses on the “benefits” and the “obligations” of being sick [14]. One of the benefits is that the person is entitled to support by others due to ill health. This situation can go on until the person is well again, and may act as a disincentive for getting well again if there is no reasonable chance of return to work. The obligations of being sick, on the other hand, imply that the period of sickness shall be considered as temporary and the person shall do everything in his or her power to get well again [14]. But then there must be something that is worth being healthy for, such as a reasonable chance to get back into the labour market. In a study from the TCO, an umbrella organization for white-collar unions in Sweden, a mere 7 % of supervisors were positive about hiring a person who has been on long-term sick leave [15]. Structural factors on the labour market may act as a hindrance for persons on sick leave returning to work. Reasons may include slimmed organizations where every employee has to work at full pace, and where support to persons with disabilities is hard to give. Previous unemployment has been seen to act as a stigma, and is often a signal to employers that productivity may be low for this person [16]. The same is probably true for sick leave and staying on sick leave may then be the most rational decision. Individuals with functional disabilities has been shown to have an increased risk of unemployment [17]. Therefore it may be a selection of unhealthy persons into long-term unemployment. Unemployment may in turn lead to further sickness due to marginalisation both in labour market and in society.

Motivation and self-efficacy are strong factors as to why people return to work. In a study from Sweden, only 4 % of individuals who believed that they were too sick to work were at work 1.5 years later [18]. When the self-efficacy is low the probability of return to work is decreased [19]. It may also be hard to return to work before complete recovery if there are no possibilities for adjustment of work tasks and working time. Persons with a high score on adjustment latitude will have a better chance to return to work earlier in the sick spell [20]. The crucial point is also to stay at work for a longer period once a person has returned to work. Often the support from health care and occupational care is reduced as soon as the person has returned to work. A study from the Netherlands shows that an intervention aiming, in a structured manner, to identify barriers and try to overcome them after a person has returned to work has been seen to decrease the likelihood of recurrent spells of sick leave [21]. The involvement of the employer in the rehabilitation process has a great impact for the length of the sickness period [22].

Loisel et al. studied patients with low back pain and suggest that illness in itself is not the decisive factor for sick leave; rather, there are other factors like problems at work, problems in relations, problems with coping, etc. that are decisive. Also, the regulations and easy access to benefits may have an impact [23]. About 70 % of all individuals in Sweden have some form of illness (i.e. subjective symptoms), and about 40 % have some form of disease (i.e. a diagnosed disease). The sick leave rate is, however, much lower [24]. In Sweden in a recent randomized controlled trial (RCT), an intervention with cognitive behavioural therapy reduced subjects’ psychological symptoms and increased their wellbeing, but did not reduce the days of sick leave [2]. This finding should sound the alarm for administrators and policy makers. It is not always enough to get well again, because something seems to happen to the employability of persons who go on sick leave.

**Fig. 5:**
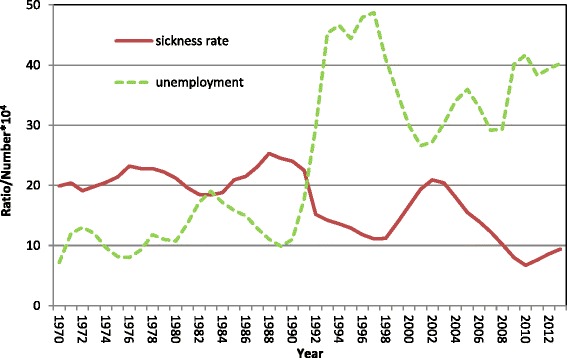
Sickness rate^1^ and unemployment^2^ in Sweden, 1970–2012. ^1^Days of sickness benefit per insured person. ^2^Yearly averages of number of people x 10^4^, taken from weekly surveys. From October 2007, job-seeking full-time students were also included. Source: Unemployment data are taken from the public statistical database at Statistics Sweden (www.scb.se); data on sickness rate are derived from the Swedish Social Insurance Agency (www.fk.se)

Historically, there is a relation between sickness rate and unemployment. When there is an increase in sickness rate, there is a decrease in unemployment, and vice versa (Fig. 5) [25, 26]. Regulations and practice, for example ceilings for replaced income loss, time limits, monitoring habits, etc. may have an impact on which insurance individuals end up with [25, 26]. In Sweden, it is financially beneficial to switch from unemployment benefit to sickness benefit, but not the other way around, even if regulations have tried to align the compensation between the two insurances. Sickness absence can to some extent be hidden unemployment [26]. There is also a bidirectional causal relationship between illness and unemployment [4, 27]. A Swedish study has found that there may be large differences in the distribution between unemployment, sick leave and disability pension between municipalities, although the total sum of all may be nearly the same [12]. Few studies have, however, addressed this complexity when assessing the social consequences of sickness absence.

### Gender

Women’s share of total days of sick leave has increased steadily during the last decades. Reasons for this may be inequality between the sexes and that women have more responsibility at home, etc. while working full-time. Another reason may be the differentiated labour market in Sweden, where women more often work in the public sector doing physically and also psychologically more demanding work [28]. Women in gender-segregated workplaces, both male- and female-dominated, have higher risk of sick leave compared with men [29].

### Education

Educational level seems to be important with regard to sick leave and the associated risk of future work absence. In our study, this applied especially to shorter periods or shorter repeated spells of sickness absence. In our cohort, persons with lower education were over-represented in the group of persons on sick leave. Individuals with a university education, but also individuals with an upper secondary school education, may be better able to adjust their workload and working time in periods of sickness compared with persons with elementary schooling only. For instance, they may be able to work from home and catch up on work by making up for lost time on another day as their adjustment latitude is higher. Persons with lower education in general have manual jobs and, hence, fewer possibilities to work from home [30]. With longer periods or spells of sick leave, educational level did not moderate future work absence and the educational categories in our study seemed to be more equal.

### Country of origin

Immigrants on sick leave up to 29–59 days in 1993 had higher work absence compared with native Swedes. For longer periods of sick leave, there were no differences. Reasons to differences in later work absence may be differences in health between immigrants and natives, as suggested in a study from the Netherlands [31]. Disability pension claims have been more common among immigrants than among the native population, both in Sweden and in Norway [8, 9]. Socio-economic factors have been put forward as a reason for immigrants to have more days of hospitalization compared with native Swedes [32]. In a study from Canada, immigrants often work well below their formal competence, if they work at all. This, too, is a stressor leading to health issues [33]. There may furthermore be structural discrimination, which can double the burden among immigrants on sick leave [34]. Notably in our study, for sick leave periods over 59 days there were no differences between immigrants and native-born Swedes. One reason for this may be that there were few persons in each group who took sick leave for >120 days, which gave low power to the calculations in the groups. Another reason may be that the cohort was young, and the majority of the immigrants had immigrated at school age. According to a Swedish study, arrival in Sweden at an early age is associated with better self-rated health [35]. There may also be a so-called “healthy migrant effect” of migration, indicating a selection of healthy individuals leaving the native country [36].

### Strengths and weaknesses

Sweden has a well-developed register system, which allows a comprehensive longitudinal approach. This study had a large study population and was a total investigation of immigrants. We followed a cohort for 15 years and measured the number of days of work absence year by year. As with all registers there are, however, some shortcomings. Data on educational background were self-reported if an immigrant had never participated in the Swedish school system. The information was therefore more uncertain in this group and missing values were more common. Persons may also be sick and unemployed without getting benefits from the Swedish Social Insurance Agency. They may be supported by relatives and/or they may not qualify for sickness or unemployment benefits. This group has not been included in this study, which may have led to an underestimation of the risk of being on sick leave.

## Conclusion

There is an association between claimed sick leave and subsequent work absence showing a steady increase of later work absence for every increase in length of sick leave. Also short periods of claimed sick leave increased subsequent work absence. We cannot, however, conclude that sick leave is the cause for later work absence, but sick leave should not be used without considering that sick leave for some persons may lead to unwanted effects on later attachment to the labour market. Some groups in society may have a higher risk of ending up outside the labour market. Women, persons with low education and immigrants had more work absence for every given exposure of sick leave. In targeting programmes and interventions for persons on sick leave, it is important to identify groups that are more vulnerable to adverse effects of sick leave.

## Availability of data and materials

The datasets supporting the conclusions of this article are available at request at Occupational and Environmental Medicine at the Department of Medical Sciences at Uppsala University, Ulleråkersvägen 40 in Uppsala.

## Appendix 1

### Statistics

**Table 2:** Zero Inflated Negative Binomial model of total work absence 1994–2008. IRR for women versus men

*Sick leave 1993*	*Woman Mean(SD)*	*Man Mean(SD)*	*Adjusted IRR Woman vs Man*	*95 % CI*	*p-value*
0	*n =* 59814 380 (620)	*n =* 59876 241 (507)	1.20	1.18–1.22	<.0001
1–7	*n =* 3241 626 (844)	*n =* 1599 436 (737)	1.35	1.24–1.47	<.0001
8–14	*n =* 1440 723 (957)	*n =* 863 423 (698)	1.52	1.35–1.70	<.0001
15–28	*n =* 1600 763 (1003)	*n =* 905 548 (895)	1.28	1.14–1.43	<.0001
29–59	*n =* 1678 919 (1106)	*n =* 760 580 (963)	1.36	1.21–1.53	<.0001
60–89	*n =* 718 1074 (1225)	*n =* 312 671 (1061)	1.50	1.25–1.79	<.0001
90–119	*n =* 424 1157 (1246)	*n =* 166 874 (1296)	1.26	0.99–1.61	0.0649
120–149	*n =* 201 1596 (1473)	*n =* 106 851 (1143)	1.81	1.32–2.48	0.0002
150–179	*n =* 145 1478 (1472)	*n =* 82 1111 (1388)	1.30	0.91–1.85	0.1493
180–239	*n =* 181 1998 (1620)	*n =* 111 1251 (1451)	1.50	1.10–2.04	0.0104
240–299	*n =* 114 1984 (1602)	*n =* 65 1858 (1724)	1.13	0.76–1.68	0.5459
330–364	*n =* 137 2421 (1833)	*n =* 116 2120 (1740)	1.19	0.87–1.64	0.2812
365	*n =* 130 3604 (1919)	*n =* 113 3187 (2010)	1.22	0.88–1.69	0.2380

Adjusted for age, education, origin and county, IRR (Incidence-rate ratio) from the negative binominal part of the model

**Table 3:** Zero Inflated Negative Binomial model of total work absence 1994–2008. IRR for immigrant versus native Swede

*Sick leave 1993*	*Immigrant Mean(SD)*	*Native Swede Mean(SD)*	*Adjusted IRR Immigrant vs Native Swede*	*95 % CI*	*p-value*
*Sick leave 1993*	*Immigrant Mean(SD)*	*Native Swede Mean(SD)*	*Adjusted IRR Immigrant vs Native Swede*	*95 % CI*	*p-value*
0	*n =* 12059 555 (798)	*n =* 107631 283 (532)	1.59	1.54–1.63	<.0001
1–7	*n =* 542 759 (884)	*n =* 4298 539 (803)	1.29	1.15–1.46	<.0001
8–14	*n =* 226 1033 (1136)	*n =* 2077 564 (836)	1.63	1.36–1.97	<.0001
15–28	*n =* 315 894 (1007)	*n =* 2190 655 (962)	1.32	1.12–1.54	0.0007
29–59	*n =* 318 1112 (1165)	*n =* 2120 768 (1053)	1.31	1.12–1.53	0.0007
60–89	*n =* 150 1158 (1270)	*n =* 880 917 (1175)	1.12	0.89–1.41	0.3448
90–119	*n =* 84 1546 (1441)	*n =* 506 1000 (1219)	1.40	1.03–1.90	0.0294

Adjusted for age, education, origin and county, IRR (Incidence-rate ratio) from the negative binominal part of the model

**Table 4:** Zero Inflated Negative Binomial model of total work absence 1994–2008. IRR for education

				*University vs Upper secondary*			*Elementary vs Upper secondary*		
*Sick leave 1993*	*Elementary Mean(SD)*	*Upper secondary Mean(SD)*	*University Mean(SD)*	*Adjusted IRR*	*95 % CI*	*p-value*	*Adjusted IRR*	*95 % CI*	*p-value*
0	*n =* 11847 626 (901)	*n =* 79248 300 (542)	*n =* 28595 209 (399)	0.75	0.73–0.76	<.0001	1.81	1.76–1.86	<.0001
1–7	*n =* 770 942 (1063)	*n =* 3601 510 (751)	*n =* 469 346 (609)	0.73	0.63–0.83	<.0001	1.76	1.58–1.95	<.0001
8–14	*n =* 406 972 (1149)	*n =* 1683 558 (811)	*n =* 214 340 (584)	0.63	0.52–0.77	<.0001	1.60	1.38–1.85	<.0001
15–28	*n =* 480 1067 (1239)	*n =* 1793 618 (881)	*n =* 232 419 (773)	0.67	0.55–0.81	<.0001	1.68	1.46–1.92	<.0001
29–59	*n =* 499 1152 (1244)	*n =* 1747 744 (1017)	*n =* 192 568 (920)	0.75	0.61–0.92	0.0069	1.44	1.26–1.65	<.0001
60–89	*n =* 221 1513 (1445)	*n =* 730 797 (1050)	*n =* 79 819 (1190)	1.02	0.75–1.40	0.8798	1.80	1.47–2.20	<.0001
90–119	*n =* 131 1387 (1413)	*n =* 411 1029 (1234)	*n =* 48 651 (892)	0.68	0.45–1.02	0.0621	1.32	1.02–1.72	0.0346

Adjusted for age, education, origin and county, IRR (Incidence-rate ratio) from the negative binominal part of the model
